# Differences in Aortic Valve and Left Ventricular Parameters Related to the Severity of Myocardial Fibrosis in Patients with Severe Aortic Valve Stenosis

**DOI:** 10.1371/journal.pone.0170939

**Published:** 2017-01-27

**Authors:** Inyoung Song, Sung Min Ko, Jeong Geun Yi, Hyun Keun Chee, Jun Seok Kim

**Affiliations:** 1 Department of Radiology, Konkuk University Medical Center, Konkuk University School of Medicine, Seoul, Korea; 2 Department of Thoracic Surgery, Konkuk University Medical Center, Konkuk University School of Medicine, Seoul, Korea; Brigham and Women's Hospital, Harvard Medical School, UNITED STATES

## Abstract

**Objective:**

This study investigated the morphological and functional characteristics of the aortic valve and the left ventricular (LV) systolic functional parameters and myocardial mass related to the severity of myocardial fibrosis (MF) in patients with severe aortic valve stenosis (AS).

**Materials and Methods:**

We retrospectively enrolled 81 patients (48 men; mean age: 59±12 years) with severe AS who underwent transthoracic echocardiography (TTE), cardiac computed tomography (CCT), and cardiovascular magnetic resonance (CMR) within 1 month and subsequent aortic valve surgery. Degree of MF was determined on delayed contrast-enhanced CMR with visual sub-segmental analysis-based quantification and was classified into three groups (no, mild, and severe) for identifying the differences in LV function and characteristics of the aortic valve. One-way ANOVA, Chi-square test or Fisher’s exact test were used to compare variables of the three groups. Univariate multinomial logistic regression analysis was performed to determine the association between the severity of MF and variables on imaging modalities.

**Results:**

Of 81 patients, 34 (42%) had MF (mild, n = 18; severe, n = 16). Aortic valve calcium volume score on CCT, aortic valve area, LV mass index, LV end-diastolic volume index on CMR, presence of mild aortic regurgitation (AR), transaortic mean pressure gradient, and peak velocity on TTE were significantly different among the three groups and were associated with severity of MF on a univariate multinomial logistic regression analysis. Aortic valve calcium grade was different (*p* = 0.008) among the three groups but not associated with severity of MF (*p* = 0.375).

**Conclusions:**

A multi-imaging approach shows that severe AS with MF is significantly associated with more severe calcific AS, higher LV end-diastolic volume, higher LV mass, and higher prevalence of mild AR.

## Introduction

Aortic valve stenosis (AS) is the most common valvular heart disease (VHD) requiring valve replacement and increases in prevalence with advancing age [1–3]. AS leads to increased left ventricular (LV) afterload and causes compensatory LV hypertrophy to minimize wall stress and maintain cardiac output. LV hypertrophy can cause reversible myocardial ischemia before deteriorating to irreversible myocardial injury such as interstitial myocardial fibrosis (MF) that may lead to LV systolic and diastolic dysfunction [4]. Several studies have demonstrated that early surgical treatment for patients with asymptomatic severe AS improves clinical outcomes compared to patients that have become symptomatic from delayed surgical treatment [5]. However, until now, definite surgical treatment criteria for patients with asymptomatic AS have not been well established [6].

Recent studies have shown that focal MF appears as a diverse pattern of midwall delayed enhancements on delayed contrast-enhanced cardiovascular magnetic resonance (DCE-CMR) images in patients with severe AS [7–9]. The size of the delayed enhancement on DCE-CMR correlates well with the amount of MF at a histologic examination [8]. Given that MF is associated with more severe AS and a worse long-term outcome after aortic valve replacement surgery [8,10], early detection of MF could prove to be important in improving patient prognosis.

Several studies have investigated differences in the characteristics of AS and parameters of LV dysfunction according to the presence or absence of MF in patients with aortic valve dysfunction [8,11]. However, there is a lack of studies that comprehensively compare aortic valve and LV parameters using transthoracic echocardiography (TTE), cardiac computed tomography (CCT), and CMR with the severity of MF as assessed by DCE-CMR in patients with severe AS. Therefore, the purpose of this study was to compare the morphological and functional characteristics of aortic valve and the LV systolic functional parameters and myocardial mass using multiple imaging modalities related to the severity of MF in patients with severe AS.

## Materials and Methods

### Study population

The retrospective study was approved by the Ethics Committee and Institutional Review Board of Konkuk University Medical Center (KUH1140045). Informed consent was exempted. A computerized search of medical and radiological records from January 2009 and December 2012 identified 116 patients with severe AS, diagnosed on TTE, who underwent CCT and CMR within 4 weeks, and without interval change in clinical status or cardiovascular event and subsequent aortic valve surgery. Severe AS on TTE was defined as peak aortic valve velocity ≥4 m/s, mean pressure gradient ≥40 mmHg, or aortic valve area (AVA) ≤1 cm^2^, or any combination [3]. Thirty-five patients were excluded: 13 patients did not undergo DCE-CMR, 12 patients had chronic myocardial infarction detected by DCE-CMR and previous history of myocardial infarction, and 10 patients had a concurrent moderate or severe degree of other VHD including aortic valve regurgitation (AR), mitral regurgitation, or mitral stenosis. We also included patients who had mild AR in this study. Finally, 81 patients with severe AS (with no or mild AR) were enrolled, and their baseline clinical characteristics and detailed information on surgery were determined from the medical and radiological records.

### CCT scan protocol

All CCT examinations were performed using a dual-source CT scanner (Somatom Definition, Siemens Medical Solutions, Forchheim, Germany). Data acquisition was performed in the craniocaudal direction with a detector collimation of 2 × 32 × 0.6mm, a slice acquisition of 2 × 64 × 0.6mm, a gantry rotation time of 330ms, a pitch of 0.20–0.43 adapted to HR, a tube voltage of 120kV for calcium score and 100 or 120kV for coronary CT angiography (CCTA), and a tube current-time product of 80mAs per rotation for calcium scoring and 330mAs per rotation for CCTA. A non-enhanced electrocardiography (ECG)-gated CT scan, prospectively triggered at 75% of the R-R interval, was performed to measure the coronary artery and aortic valve calcium scores. For the CCTA, ECG-based tube current modulation was implemented, except for patients with mean HRs >80 beats per minute or those with arrhythmia.

Contrast agent application was controlled by a bolus tracking technique. A Stellant D dual-head power injector (Medrad, Indianola, PA, USA) was used for all CT examinations to administer a three-phase bolus at a rate of 4.5mL/s. First, 70–80mL of undiluted contrast media (Iopromide, Ultravist 370mg I/mL, Bayer-Schering Pharma, Berlin, Germany) was administered and then 45mL of a mixture of 70% contrast and 30% saline was administered with a saline chaser for CCTA.

### CCT image analysis

Analysis of CCT and CMR images was based on the consensus of two radiologists who were blind to patient clinical data, including all clinical findings, history, and TTE results. The CCT image quality was classified using a 4-point subjective ranking scale as follows: (1) bad; (2) poor, but diagnostic; (3) good; and (4) excellent.

Ten transaxial data sets were reconstructed with retrospective ECG gating at 10% steps, from 0–90% of the R-R interval for each patient to assess aortic valve morphology and function. CCTA data sets were then transferred to an external workstation (Vitrea 2, Vital Images, Plymouth, MN, USA) and reviewed by applying multiplanar reformations and a four-dimensional cine technique. Post-processing included both static and cine images of the aortic valve in double-oblique short-axis planes. For the morphological aspects of the aortic valve, several cross-sectional transverse images of the aortic valve during early-systole and mid-diastole were reconstructed.

Aortic valve calcium volume score and coronary artery calcium score were evaluated using CaScore software (Siemens Medical Solutions). The aortic valve calcification grade was categorized as absent, mild, moderate, or severe as described by Rosenhek et al. [12]. The presence of coronary artery disease was defined as at least one significant coronary stenosis (≥50% lumen diameter reduction) using dedicated vessel analysis software (Vitrea 2, Vital Images, Plymouth, MN, USA).

### CMR examination

CMR exams were performed on a Signa HDxt 1.5-T system (GE Healthcare, Waukesha, WI, USA) or a Magnetom Skyra 3.0-T system (Siemens) within 7 days after the CCT examination. Aortic valve and short-axis cine images were acquired with gradient echo fast imaging employing steady-state free precession sequence on a 1.5-T scanner (repetition time 3.54ms; echo time 1.54ms; flip angle 45°; slice thickness 5mm without interslice gaps; field of view 36 × 36cm; matrix 224 × 224; and pixel size 0.16 × 0.16cm) and a 3.0-T scanner (repetition time 39.24ms; echo time 1.43ms; flip angle 54°; slice thickness 6mm without interslice gaps; field of view 34 × 28cm; matrix 208 × 139; and pixel size 0.16 × 0.16cm). The imaging plane of the aortic valve was defined by the acquisition of a systolic three-chamber view and an oblique coronal view of the aortic valve and proximal aorta. The subsequent slices were defined parallel to the valvular plane.

Ten minutes after injecting 0.2 mmol/kg gadopentetate dimeglumine (Magnevist, Bayer, Berlin, Germany) or gadoterate meglumine (Dotarem, Guerbet, France), delayed enhancement images were acquired in two long axes and 10–11 short axes using a 1.5-T system with a phase sensitive myocardial delayed enhancement sequence (repetition time 5.7ms; echo time 2.6ms; flip angle 25°; inversion time individually adjusted; slice thickness 8mm; field of view 36 × 36cm; matrix 200 × 200) or using a 3.0-T system with phase sensitive inversion recovery sequence (repetition time 5.18ms; echo time 1.96ms; flip angle 20°; slice thickness 8mm; field of view 350 × 262mm; matrix 256 × 192).

### CMR image analysis

**Fig 1:**
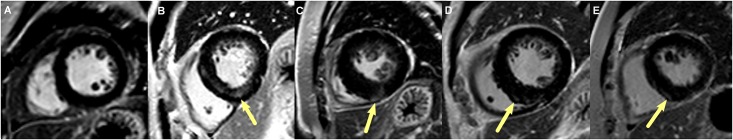
Images of variable patterns of delayed contrast enhancement on myocardium. **a** No delayed contrast enhancement on myocardium is defined as the absence of myocardial fibrosis. The patterns of myocardial fibrosis are diffuse (arrow, **b**), patchy (arrow, **c**), nodular (arrow, **d**), or linear (arrow, **e**).

Cine images of CMR were used for the assessment of LV end-diastolic volume (LVEDV), LV ejection fraction (LVEF), LV mass, and AVA using a cardiac dedicated workstation (ArgusVF, Siemens Medical Solutions). LVEDV index and LV mass index of each patient were calculated by LVEDV and LV mass divided by body surface area (m^2^) respectively. AVA was measured at cross-sectional planimetric images during systole by drawing the region of interest. The presence of focal MF on DCE-CMR was defined as linear, nodular, patchy or diffuse patterns of delayed contrast enhancement (Fig 1) [9].

**Fig 2:**
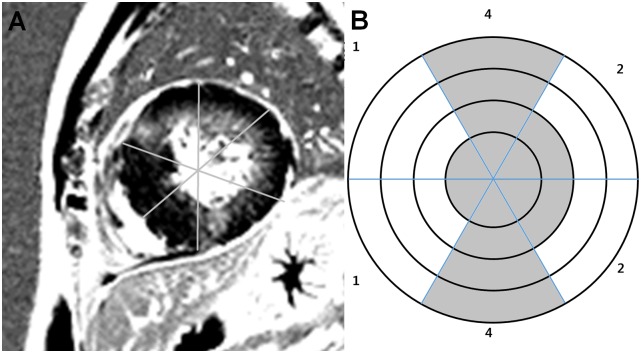
Example of sub-segmental scoring model used for visual coding of focal myocardial fibrosis location. **a** Cardiac magnetic resonance demonstrated extensive delayed contrast enhancement in mid-ventricular short axis image. **b** Mid-ventricular segments are divided into four transmural zones and coded according to transmural extent of delayed contrast enhancement.

Degree of MF was determined on DCE-CMR with visual sub-segmental analysis (VSSA)-based quantification [13]. This approach estimates the percent of transmural extent of the myocardial-delayed hyperenhancement for each of the 17 segments according to the AHA recommendations [14]. A score ranging from 0 to 4 was attributed to all of the segments according to the depth of MF: score 0 = 0%, 1 = 1–25%, 2 = 26–50%, 3 = 51–75% and 4 = 76–100% (Fig 2). All 17 scores were summed and multiplied by 1.47% for calculating total percent LV MF volume [13].

### TTE examination

We reviewed the electronic medical records of each patient’s echocardiography reports to collect findings including LVEF (%), aortic valve mean pressure gradient (mmHg), AVA (cm^2^), peak aortic valve velocity (m/s), and the presence of AR. Mild AR on TTE was defined as a proximal regurgitation jet width of <25% of the LV outflow tract, vena contracta length of <0.3 cm, and a pressure half-time of >500ms [3]. Aortic valve morphology and the presence and severity of AS were recorded by cardiologists. Two-dimensional TTE was performed with a Vivid 7 device (GE Healthcare) and an Acuson Sequoia C512 apparatus (Siemens) with 2.5–3.5 MHz imaging transducers.

### Statistical analysis

In descriptive statistical analysis, continuous variables were expressed as mean ± standard deviation. Categorical variables were described as percentage or frequency. Descriptive statistics for each variable are reported in tables. A one-way ANOVA was used to compare continuous variables of the three groups. The Chi-square test or Fisher’s exact test was also used to compare categorical variables of the three groups. Univariate multinomial logistic regression analysis was performed to determine the association between the severity of MF and variables on imaging modalities. Statistical analyses were performed using SPSS 17.0 (SPSS Inc., Chicago, IL, USA). Statistical significance was considered when the *p* value was less than 0.05.

## Results

### Patient characteristics

**Fig 3:**
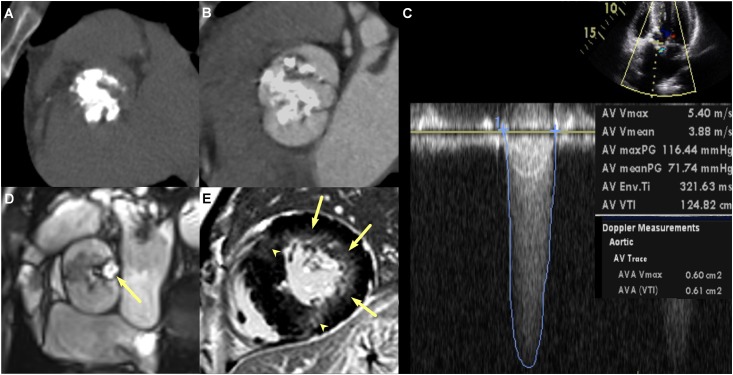
Multi-modalities images of a 45-year old man with severe aortic stenosis (AS), mild aortic regurgitation, and myocardial fibrosis (MF). **a** and **b** Cardiac computed tomography images demonstrate calcified AS (aortic valve calcification grade 4, aortic valve calcium score 7650, and aortic valve area 0.83cm^2^) and bicuspid aortic valve without raphe. **c** Transthoracic echocardiography image demonstrate severe AS (aortic valve area 0.61cm^2^), peak aortic velocity 5.4m/sec, and mean pressure gradient 71.7mmHg. **d** and **e** Cardiac magnetic resonance images demonstrated severe AS (aortic valve area 0.71cm^2^, arrow in **d**), left ventricular (LV) hypertrophy, LV end-diastolic volume index 84ml/m2, LV end-diastolic mass 268g, LV ejection fraction of 56%, presence of MF appearing delayed hyperenhancement in the subendocardial region of the lateral wall (arrows, **e**) and in the transmural region of the anterior and inferior wall of the mid-ventricular myocardium (arrowheads, **e**).

Clinical characteristics of patient are summarized in Table 1. The study population consisted of 48 men and 33 women (mean age 59.1 ± 12.4 years). Dyspnea was the major limitation. Forty-eight (59%) patients had mild AR. Among the 33 (41%) patients with isolated severe AS, one had mild mitral regurgitation. All patients underwent open-heart surgery with aortic valvuloplasty [15]. Twenty-eight (35%) patients had a tricuspid aortic valve and 53 (65%) patients had a bicuspid aortic valve according to intraoperative findings. Clinical characteristics were not significantly different among the three groups. Thirty-four (42%) patients showed LV delayed hyperenhancement suggestive of MF on DCE-CMR (Fig 3). Using VSSA-based quantification, the mean and median total MF volume of the 34 patients were 29.5 ± 20% (range 2.9–80.9%) and 27.9% of the LV mass, respectively. Using 30% of MF volume as a cut-off value for MF severity, 18 patients were assigned to the group with mild MF and 16 patients to the group with severe MF. Patients with severe MF exhibited higher MF volume than those with mild MF (46.7 ± 14.6% vs. 14.3 ± 8.9%, *p*<0.001).

**Table 1:** Clinical characteristics of patients with severe aortic valve stenosis (n = 81) according to the severity of myocardial fibrosis.

Characteristics	No MF (n = 47)	Mild MF (n = 18)	Severe MF (n = 16)	*p* value
**Age** (yrs)	60.2 ± 12.4	57.8 ± 12.7	57.5 ± 12.5	0.650
**Male**	24 (51%)	12 (67%)	12 (75%)	0.187
**BAV**	28 (60%)	15 (83%)	10 (63%)	0.190
**BMI (kg/m**^**2**^**)**	27.3 ± 7.5	26.4 ± 5.6	28.2 ± 8.2	0.907
**Chest pain**	18 (38%)	7 (39%)	1 (7%)	0.060
**Dyspnea**	23 (49%)	8 (44%)	11 (69%)	0.303
**Syncope**	2 (4%)	0 (0%)	2 (13%)	0.275
**Clinical history**				
Diabetes mellitus	8 (17%)	3 (17%)	4 (25%)	0.734
Hyperlipidemia	8 (17%)	4 (22%)	6 (38%)	0.261
Hypertension	21 (45%)	7 (39%)	5 (31%)	0.630
Smoking	9 (19%)	5 (28%)	7 (44%)	0.158
CAD	10 (21%)	7 (39%)	4 (25%)	0.325

Values are number or mean ± standard deviation. MF myocardial fibrosis, BAV bicuspid aortic valve, BMI body mass index, CAD coronary artery disease

### CCT findings

All CCT examinations were of diagnostic image quality for assessing coronary arteries and valves. The quality of most CCT images was excellent (n = 76, 94%), and others had good or fair image quality (n = 4 and 1, respectively). The mean patient radiation dose was 10.2 ± 0.7mSv on dual-source CT (including calcium scoring and CCTA and with the use of a conversion coefficient for the chest of κ = 0.017mSv∙ mGy^−1^∙cm^−1^). The average HR during CCT was 66.6 ± 2.4bpm.

CCTA identified 21 patients with significant coronary stenosis (no MF, n = 10; mild MF, n = 7; and severe MF, n = 4) without a statistically significant difference among the three groups (*p* = 0.325). Patients with severe MF had significantly higher aortic valve calcium volume scores (3544 ± 2336mm^3^, *p* = 0.002) than those with mild MF (2404 ± 1412mm^3^) and no MF (1660 ± 1091mm^3^). In addition, the grade of aortic valve calcification was significantly higher (*p* = 0.008) in the group with severe MF than in the group with mild MF and the group with no MF.

### CMR findings

AVA obtained with CMR planimetry (0.78 ± 0.14cm^2^) was not significantly different (*p* = 0.84) from that computed with TTE measurements (0.78 ± 0.15cm^2^). There was good correlation between AVAs measured with CMR and TTE (r = 0.64, *p*<0.0001). Patients with severe MF had significantly smaller AVA (0.70 ± 0.15 cm^2^, *p* = 0.011) than those with mild MF (0.75 ± 0.15cm^2^) or with no MF (0.83 ± 0.13cm^2^). LV mass index (105.3 ± 35.1g/m^2^), and LVEDV index (94.9 ± 17.2ml/m^2^) were significantly higher in patients with severe MF than those with mild MF (76.6 ± 29.3g/m^2^ and 91.7 ± 23.6ml/m^2^, respectively) or with no MF (63.3 ± 17.8g/m^2^ and 79.3 ± 19.0ml/m^2^, respectively). LVEF was not significantly different among the three groups (Table 2).

**Table 2:** Differences of multimodality imaging findings according to the severity of MF.

Characteristics	No MF (n = 47)	Mild MF (n = 18)	Severe MF (n = 16)	*p* value
**CCT**				
AV Ca score	1660 ± 1092	2405 ± 1412	3544 ± 2336	0.002
AV Ca grade				0.008
Grade 1	1 (2%)	1 (6%)	0 (0%)	
Grade 2	17 (36%)	3 (17%)	0 (0%)	
Grade 3	19 (41%)	5 (28%)	7 (44%)	
Grade 4	10 (21%)	9 (50%)	9 (56%)	
**CMR**				
LVEDV index (ml/m^2^)	79.3 ± 19.0	91.7 ± 23.6	94.9 ± 17.2	0.007
LVEF (%)	68.6 ± 11.8	66.1 ± 10.9	61.2 ± 16.8	0.253
LV mass index (g/m^2^)	63.3 ± 17.8	76.6 ± 29.3	105.3 ± 35.1	< 0.001
AVA (cm^2^)	0.83 ± 0.13	0.75 ± 0.15	0.70 ± 0.15	0.011
**TTE**				
AVA (cm^2^)	0.81 ± 0.14	0.76 ± 0.16	0.72 ± 0.19	0.062
Transaortic mean PG (mmHg)	50.5 ± 16.9	56.6 ± 12.4	65.0 ± 23.4	0.020
Transaortic peak velocity (m/sec)	4.5± 0.71	4.9 ± 0.63	5.1± 0.8	0.006
Mild AR	20 (43%)	14 (78%)	14 (88%)	0.001

Values are number or mean ± standard deviation

AV aortic valve, EDV end-diastolic volume, EF ejection fraction, LV left ventricular, MF myocardial fibrosis, AVA aortic valve area, AR aortic regurgitation, PG pressure gradient, TTE transthoracic echocardiography, CCT cardiac computed tomography, CMR cardiovascular magnetic resonance

### TTE findings

In contrast to the CMR finding, continuity equation-derived AVA measured by TTE was not different among the three groups (*p* = 0.062). However, patients with severe MF had significantly higher transaortic mean pressure gradient (65.0 ± 23.4mmHg), transaortic peak velocity (5.1 ± 0.8m/s), and higher prevalence of mild AR (88%) than those with mild MF (56.6 ± 12.4mmHg, 4.9 ± 0.63m/s, and 78%, respectively) or with no MF (50.5 ± 16.9mmHg, 4.5± 0.71m/s, and 43%, respectively).

### Univariate multinomial logistic regression analysis

Univariate multinomial logistic regression analysis identified the following significant variables among the three groups (all of the *p* values were less than 0.05): aortic valve calcium volume scores on CCT, AVA, LV mass index, LVEDV index measured on CMR, transaortic mean pressure gradient, transaortic peak velocity, and the presence of mild AR on TTE (Table 3).

**Table 3:** Odds ratio for imaging variables for the severity of myocardial fibrosis on univariate multinomial logistic regression analysis.

Variables	Odds ratio of Severe MF vs. No MF	Odds ratio of Mild MF vs. No MF	Odds ratio of Severe MF vs. Mild MF	Overall *p* value
**CCT**				
AV Ca score	1.001*(1.000, 1.001)	1.000*(1.000, 1.001)	1.000(1.000, 1.001)	0.003
AV Ca grade				0.375
Grade 1	<0.001(<0.001, >999.99)	1.111(0.060, 20.487)	<0.001(<0.001, >999.99)	
Grade 2	<0.001(<0.001, >999.99)	0.196(0.043, 0.899)	<0.001(<0.001, >999.99)	
Grade 3	0.409(0.117, 1.428)	0.292(0.077, 1.111)	1.400(0.321, 6.109)	
Grade 4	N/A	N/A	N/A	
**CMR**				
LVEDV index (ml/m^2^)	1.042*(1.010, 1.075)	1.036*(1.005, 1.068)	1.006(0.977, 1.036)	0.019
LVEF (%)	0.958(0.918, 1.001)	0.983(0.940, 1.027)	0.975(0.928, 1.024)	0.154
LV mass index (g/m^2^)	1.059*(1.030, 1.090)	1.028*(1.002, 1.055)	1.030(1.005, 1.057)	<0.001
AVA (cm^2^)	0.002*(<0.001, 0.145)	0.013*(<0.001, 0.858)	0.117(<0.001, 16.422)	0.011
**TTE**				
AVA (cm^2^)	0.018*(<0.001, 0.939)	0.130(0.003, 5.022)	0.135(0.001, 12.849)	0.119
Transaortic mean PG (mmHg)	1.041*(1.008, 1.075)	1.022(0.990, 1.056)	1.018(0.984, 1.054)	0.046
Transaortic peak velocity (m/sec)	2.834*(1.255, 6.403)	2.250*(1.020, 4.964)	1.259(0.545, 2.912)	0.027
Mild AR	9.45*(1.926, 46.360)	4.725*(1.350, 16.535)	2.00(0.314, 12.745)	0.003

Values are number (95% confidence interval)

* Statistically significantly different with *p* < 0.05

AV aortic valve, EDV end-diastolic volume, EF ejection fraction, LV left ventricular, MF myocardial fibrosis, AVA aortic valve area, AR aortic regurgitation, PG pressure gradient, TTE transthoracic echocardiography, CCT cardiac computed tomography, CMR cardiovascular magnetic resonance

## Discussion

In the present study, using multiple imaging modalities, we found significant correlation between the severity of MF and characteristics of severe AS with each imaging modality. In patients with severe AS, without other significant valve diseases and MI, the severity of MF detected by DCE-CMR was significantly associated with aortic valve calcium score on CCT, AVA, LV mass index and LVEDV index on CMR, and transaortic mean pressure gradient, transaortic peak velocity and presence of mild AR on TTE.

The presence of MF is associated with a worse prognosis, such as worsening LV systolic function and ventricular stiffness assessed by using TTE and CMR in several heart diseases including severe AS and severe AR [8,10,16–19]. Therefore, early detection of MF is an independent predictor of survival in patients with moderate to severe AS and is of incremental value in the prognostic model to LVEF [9]. The quantitative amount of focal MF measured by DCE-CMR correlated well with the values of interstitial MF obtained by histology [20]. In the present study, we used a VSSA-based quantification method for severity of MF on DCE-CMR, which was validated in previous studies [13].

CCT allows for accurate detection, localization and quantification of aortic valve calcification [20]. However, data are limited comparing the severity of aortic valve calcification on CCT with the severity of MF on DCE-CMR in patients with severe AS. We found that calcium volume score and calcification grade of the aortic valve were significantly different among the three groups but only calcium volume score was associated with the severity of MF. Aortic valve calcification is considered along with imaging markers to predict progression of AS [21]. High calcification of the aortic valve is associated with worse morbidity and mortality in patients with AS, even with low-gradient, low-flow severe AS [9,22]. A multicenter outcome study has shown that severe aortic valve calcification provides significant additive value to the prediction of mortality under medical treatment and also independently predicts overall mortality in patients with AS [23]. Along with these prognostic values, based on our results, the quantitative assessment of aortic valve calcification on CCT may be a surrogate parameter of MF in patients with severe AS.

Severe AS is often associated with some degree of AR [24]. Honda et al. reported patients with severe AS and significant (moderate to severe degree) AR had significantly worse outcomes than patients with severe AS with no or trivial AR [25]. Significant AR leads to the degeneration of cardiomyocytes and myocardial function and exacerbates the myocardial ischemia in patients with severe AS [26–29]. In the present study, concomitant mild AR on TTE was associated with the severity of MF. However, our study does not provide conclusive evidence to support the causal relationship between mild AR and MF because there is no scientific evidence available that mild AR influences the development of MF in patients with severe AS and the percentage of patients having mild AR is too large (59%).

Continuity equation-derived AVA by 2D-TTE is usually smaller than AVA measured with 3D-TTE, CCT, and CMR by planimetry [30,31]. The orifice of the vena contracta is smaller than the actual anatomical maximum opening of the stenotic aortic valve [30]. In addition, the 2D-TTE underestimates the left ventricular outflow tract by using a single-diameter measurement assuming circular geometry when compared with CMR [32]. In our study, there was good correlation and no statistically significant difference between CMR and TTE measurements of AVA. However, only AVA measured on CMR was significantly different among the three groups and was associated with the severity of MF. CMR-derived AVA may not be as accurate as continuity equation-derived AVA by TTE in the detection of severe AS but can be used as a predictor of MF in patients with severe AS.

In the present study, positive graded association with the severity of MF was found for LVEDV index, LV mass (CMR), transaortic peak velocity, and mean aortic gradients (TTE). These parameters were significantly associated with the severity of MF. However, LVEF, as the reference standard for global LV systolic function, was similar among the three groups and was not significantly associated with the severity of MF. Our study findings are consistent with a previous study in which LVEF was preserved, but LVED diameter was increased in severe AS patients with severe MF [8].

Based on the results of this study, the incremental values of the multi-imaging approach over DCE-CMR alone, for assessment of MF in patients with severe AS, are as follows: 1. Coexisting mild AR and echocardiographic parameters of higher aortic jet velocity and mean gradient are associated with severity of MF. 2. Smaller AVA, higher LVEDV index, and higher LV mass are related to the severity of MF on CMR and may need to be incorporated into clinical management strategies. 3. Severity of aortic valve calcification as measured by CCT may be used for predicting the presence and severity of MF. A multi-modality imaging approach may help to ensure the appropriate timing of aortic valve replacement or transaortic transcatheter valvular implantation in patients with severe AS, particularly in asymptomatic moderate to severe AS or low-gradient severe AS.

The present study had several limitations. First, this study was subject to the limitations inherent in a single institution study. Second, retrospective analysis of observational data could not demonstrate how the multi-imaging approach was useful for the management of severe AS. Third, the number of cases with MF was small. Fourth, there were no referable criteria of DCE-CMR to divide patients who had severe AS and MF into mild and severe grade groups. For MF severity on DCE-CMR, we modified the cut-off value of 30% MF volume which was a moderate degree on a endomyocardial biopsy [10]. Fifth, an endomyocardial biopsy was not performed to confirm the presence or absence of MF during aortic valve surgery. Sixth, we could not evaluate the presence of diffuse MF using T1 mapping including extracellular volume evaluation because of absence of T1 mapping sequences [26]. Seventh, CMR exams were performed with two kinds of MR scanners (1.5-T and 3.0-T systems) and contrast agents (Magnevist and Dotarem). A 3.0-T MR scanner is likely to allow for better image quality and improve confidence of diagnosis. However, another previous study demonstrated no benefits for diagnosing MF with a 3.0-T compared to a 1.5-T MR scanner [33]. Finally, all CCT and CMR images were analyzed by a consensus of two radiologists. Accordingly, we did not assess intra-and inter-observer variability of CCT and CMR measurements.

In conclusion, patients with severe AS and MF are significantly associated with more severe calcific AS, higher LV end-diastolic volume, higher LV mass, and higher prevalence of mild AR. These parameters might be useful for identifying MF in patients with severe AS. However, a prospective clinical trial is warranted to confirm these findings and to determine the clinical benefits of a multi-imaging modality approach in patients with severe AS.

## Supporting Information

S1 File Dataset for all individuals. (XLS)
